# The Effects of Liquid Disinfection and Heat Sterilization Processes on Implant Drill Roughness: Energy Dispersion X-ray Microanalysis and Infrared Thermography

**DOI:** 10.3390/jcm9041019

**Published:** 2020-04-04

**Authors:** Antonio Scarano, Morena Petrini, Filiberto Mastrangelo, Sammy Noumbissi, Felice Lorusso

**Affiliations:** 1 Department of Medical, Oral and Biotechnological Sciences, University of Chieti-Pescara, 66100 Chieti, Italy; petrini.morena@gmail.com (M.P.); sammy@iaoci.com (S.N.); drlorussofelice@gmail.com (F.L.); 2 International Academy of Ceramic Implantology, Silver Spring, MD 20901, USA; 3 Department of Oral Implantology, Dental Research Division, College Ingà, UNINGÁ, Cachoeiro de Itapemirim 29312, Brazil; 4 Clinical and Experimental Medicine, Medical School, University of Foggia, Via Antonio Gramsci, 89, 71122 Foggia, Italy; filiberto.mastrangelo@unifg.it

**Keywords:** dental implant, steel drill, zirconia drill, bone osteotomy, infrared thermography

## Abstract

Background: The aim of this study is to evaluate effects on stainless steel (SS) and zirconia implant drills of 50 cycles of sterilization through different processes. Methods: A total of 24 SS and 24 zirconia drills were treated with 3 different sterilization processes: 50 cycles of immersion in glutaraldehyde 2%, 50 cycles in 6% hydrogen peroxide and 50 cycles of heat. Energy-dispersive X-ray spectroscopy (EDX) was used to compare the effect of the different treatments compared to new untreated controls. Infrared thermography was used to measure the increase of temperature during drilling on bone ribs. A scanning electron microscopy (SEM) was used to measure the roughness of the samples. Results: Zirconia drills seem not to be affected by the different treatments; no significant differences were found with EDX nor through thermography controls. SS drills were affected by the different treatments, as confirmed by the increased roughness of the SS samples after all the cycles of sterilization/disinfection, measured at SEM. On the contrary, the zirconia drills roughness was not particularly affected by the chemical and thermal cycles. Significant differences were observed regarding the temperature, between steel and zirconia drills (*p* < 0.01). Conclusions: The disinfection agents had a weak impact on the temperature changes during implant bone preparation, while heat sterilization processes had no effect on either of the drills evaluated. The disinfection agents increased the roughness of the steel drills, while they had no effect on the zirconia drills.

## 1. Introduction

Rehabilitation of edentulous patients can be successfully performed by exploiting the biologic phenomenon of osseointegration [1]. Despite many advances in techniques, materials and implant design, implant failure is a significant concern for implantology. Zirconia is a good material used in implantology for its good aesthetic properties and biocompatibility [2]. In dentistry, zirconia is largely used for implant abutment and superstructure production [3] because of its strength, durability, corrosion resistance and response to disinfection and sterilizing agents [4].

Early implant failures have been associated with endogenous host factors that could affect bone healing, such as the bone quality and quantity, the presence of systemic or metabolic diseases and smoking, but also to exogenous factors such as implant features, the surgical technique and implant site infections [5,6]. The first stage for achieving osseointegration is atraumatic implant bed preparation and the surgical technique for this usually comprehends a sequential use of drills of increasing diameters. This procedure is common and routine, however, it should be affected with attention, in order to avoid macroscopic and microscopic mistakes that could negatively influence the long-term success of the dental implant.

The heat generated during the phase of drilling determines an irreversible injury to the proteins of adjacent bone that can result in a variable layer of osteonecrosis that depends on the surgical trauma, the temperature reached and the drilling time. Ericsson et al. have shown that 30 s at 50 °C or 1 min at 47 °C are the critical parameters for bone necrosis formation [7]. Nowadays the use of digital implant planning permits to avoid macroscopic errors such as an incorrect insertion axis, injury to vascular or nerve structures; it reduces the necessity of extensive flaps and permits to evaluate the quality and quantity of bone at the site before surgery. At the same time, it allows a prosthetically driven approach, resulting in the best possible design of the prosthesis and aesthetics, optimizing the occlusion and loading [8]. It is important to highlight that, notwithstanding these multiple advantages, there is some debate in the literature about the risk of increased heat generation in the implant site with the use of surgical guidesc [9].

However, while the implant bed preparation can be affected by manual hand-pieces, there are other parameters regarding heat generation that cannot be fully controlled.

The pressure that is exerted by the operator on the drill and then on to the bone, the number of times that each drill is fully inserted and extracted from the osteotomy site and the rate of site diameter increment during drilling are all operator-dependent [10,11,12]. Other factors are related to the devices used for implant site preparation, such as the type and quantity of irrigation, the morphology of the bur, its speed and torque during drilling.

Among macroscopic features of the drill, the cutting lips, the rake angle, the flute and the helix angle can influence the magnitude of the heat generated [13,14]. Moreover, the tip geometry and the configuration of the flutes can also influence the ability of the surgeon during drilling, bur deformation and the incidence of drill-bit failure [15,16]. Also, the microscopical features of the drill play an important role: a surface with valley-shaped morphology, characterized by a lower shear contact with bone during drilling, is less subject to wear and heat generation [13].

The amount of wear of the burs, as a consequence of a great number of osteotomies, reduces their cutting ability, increases the quantity of the heat generated and the possible release of contaminants on the implant sites [9,17,18,19]. Moreover, during the drilling process, the burs are contaminated with saliva, blood and pathogens and considering their complex architecture, a thorough process of decontamination and disinfection is necessary, before sterilization, in order to eliminate the risk of cross-infection [20]. This mandatory process could lead to phenomena of corrosion, altering the surface of the burs [21]. For this reason, it is essential to manage implant drills properly and use the most efficient sterilizers. The use of disinfection/sterilization processes for implant drills is fundamental in clinical activity for many reasons. The first being to avoid the spreading of cross-infection among the patients. Secondly, it has been hypothesized that the use of aseptic techniques could also affect implant success, as recently shown by Veitz-Keenan et al. (2018) [22].

The alternative could be to use dispensable drills, but considering the high prices of Implant kits, these options are currently not convenient for clinicians and patients.

Consequently, when clinicians have to decide the protocol of disinfection/sterilization to adopt, they have to take into consideration not only the effectiveness of the product and process but also how they could affect the features of the drill.

In particular, it is fundamental to understand how many cycles can be repeated without significantly affecting some characteristics of the drills, such as their cutting ability, which consequently affects the surgical act. A review by Augustin et al. (2012) has highlighted the impact of such drill parameters, which could contribute to the increase of temperature to above 47 °C which is the threshold above which thermal osteonecrosis occurs, which contributes to screw loosening and subsequent implant failures and refractures [23].

The first hypothesis of this study is that the alterations induced by disinfection agents produce an increase in surface roughness. The second hypothesis of this study is that alterations induced by disinfection agents produce an increased temperature during implant bed preparation. The purpose of this study is to evaluate the alteration produced by liquid disinfection and heat sterilization processes on implant drills and their influence on drilling temperature in steel vs. zirconia drills.

## 2. Materials and Methods

**Figure 1:**
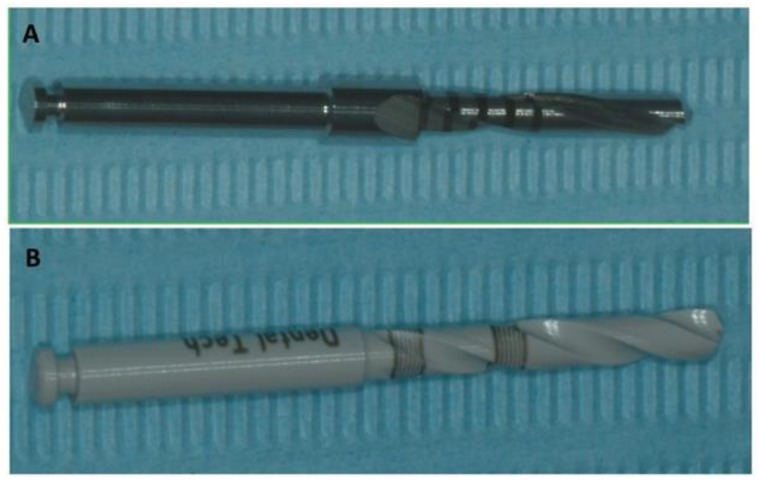
(**A**) Aspect of the stainless steel (SS) drill. **(B**) The aspect zirconia drill.

**Figure 2:**
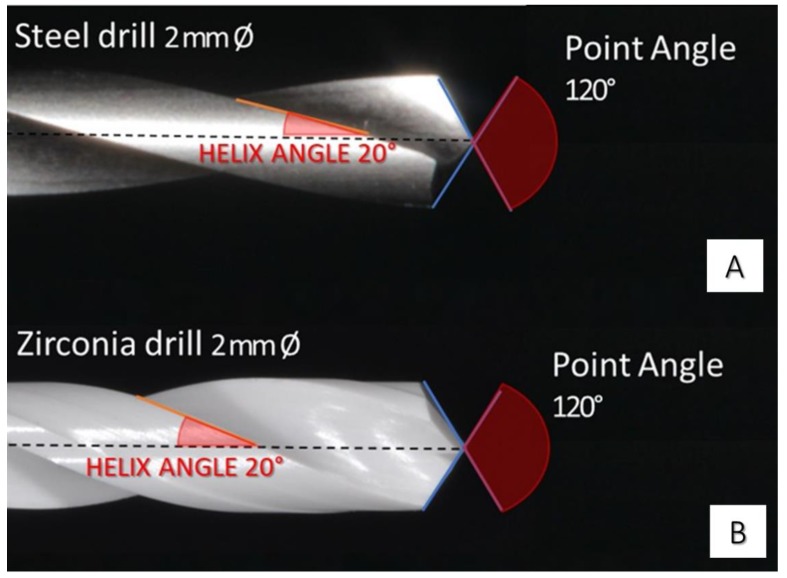
(**A**) Steel (SS) 2 mm diameter drill; point angle 120°, helix angle 20°. (**B**) Zirconia 2 mm diameter drill with positive point angle 120°, helix angle 20°.

In this study, a total of 24 drills in zirconia (Dental Tech snc Misinto, Milan, Italy) and 24 steel drills were used (Dental Tech snc Misinto, Milan, Italy), 6 drills for each group (Figure 1 and Figure 2). The sample size was calculated by using a free software (6.0 ClinCalc LLC, North Chicago, IL, USA) available on the site http://clincalc.com/stats/samplesize.aspx considering a power of 0.80, an alpha error of 5% and an effect size of 0.84, based on a previous study [19]: a total of 20 implant osteotomies on bovine ribs for each study group (total 160 sites, 5 osteotomies for each rib) were necessary to achieve statistical significance for quantitative analyses of drill surface damage.

All the drills, with the exception of Group 1 and 5 that were controls, were immersed in high protein concentration solutions (10% of fetal bovine serum) (Sigma Aldrich, Milan, Italy) phosphate-buffered saline for 10 min then they were rinsed in running water and scrubbed with a toothbrush made of nylon bristles in order to remove gross foreign material and were then subjected to the different treatments as follows and then analyzed.

Group 1: 6 New Steel drills subjected to 0 cycles of disinfection: controls.

Group 2: 6 Steel drills subjected to 50 cycles of 20 min immersion in glutaraldehyde 2% (Dimexid, Amedics Ferrara, Italy).

Group 3: 6 Steel drills subjected to 50 cycles of 20 min in hydrogen peroxide 6% (Sanibios, Noda, Balerna–Switzerland).

Group 4: 6 Steel drills subjected to 50 cycles of heat sterilization in autoclave at 134 °C for 35 min at a pressure of 1.1 bar (A-17 PLUS, ANTHOS, Imola–Italy).

Group 5: 6 new zirconia drills (Step Drill, SAFE Implant, Malaysia): controls.

Group 6: 6 zirconia drills that underwent 50 cycles of 20 min immersion in glutaraldehyde 2% (Dimexid, Amedics Ferrara, Italy).

Group 7: 6 zirconia drills (Step Drill, SAFE Implant, Malaysia) that underwent 50 cycles of 20 min immersion cleansing in hydrogen peroxide 6% (Sanibios, Noda, Balerna–Switzerland).

Group 8: 6 zirconia drills (Step Drill, SAFE Implant, Malaysia) that underwent 50 cycles of heat sterilization in autoclave at 134 °C for 35 min at a pressure of 1.1 bar (A-17 PLUS, ANTHOS, Imola– Italy).

### 2.1. Energy-Dispersive X-Ray Spectroscopy (EDX) Analysis

An FE scanning electron microscope (SIGMA HV—Carl Zeiss with Bruker Quantax 200—Z10 EDS Detector) equipped with an energy dispersive spectroscopy (EDS) was used for EDX analysis. Each sample was attached to aluminum sample holders (Stubs) using double-sided carbon disks. Each drill was analyzed in the lateral Band.

### 2.2. Infrared Thermography

The drills of each group were used for one implant bed preparation. A 14-bit digital infrared camera (FLIR SC3000 QWIP, Flir Systems, Danderyd, Sweden) was used to acquire thermal images series during implant site preparation on bone ribs at a speed of 800 rev/min with a constant load of 20 N/cm. Six drills for each group were used. During drilling the bone ribs were always in a thermostat-controlled saline bath (37.0 °C ± 0.1 °C) leaving 3 mm of bone emerged out of solution at a rate of 40 mL/min at room temperature.

According to a previous study [19], the thermography measurements were recorded in a climate-controlled laboratory conditions (temperature: 24 °C, relative humidity: 50% ± 5% and no direct ventilation on the bone) prior a calibration setting of the camera to a temperature-gauged blackbody and plotting the temperature over 5 min according to the manufacturer specifications.

The infrared thermographic scan was positioned at a focal distance of 1 m from the specimens. Temperature changes in cortical bone and in the apical portion of the drills were determined by infrared thermography, using images extrapolated by a video of the implant drilling via a dedicated software (FLIR Reporter version 8.5, Danderyd, Sweden) (Table 1).

### 2.3. Scanning Electron Microscopy (SEM)

Implant drills were assessed before and after disinfection by a scanning electron microscopy (SEM), JSM-6480LV; Jeol, Tokyo, Japan), that is characterized by a solid-state backscattered detector operated in 20 kV accelerating voltage. The drills were glued to a disc of carborundum for observation by SEM. Five flat areas of 200 μm to 150 μm in diameter were evaluated for both drills and an image in TIF format was created. The drills in zirconia and in steel were studied for the analysis of the surface topography by SEM. Two stereographic images with different tilt angles were used to calculate a 3D image of the surface. These 3D images were used for surface analysis. Surface topography parameters were obtained using the instrument’s software. For evaluation of the surface roughness, the different roughness parameters were recorded in each of the 48 drills. Means for each parameter were calculated and average Ra: arithmetical mean deviation, Rq: root mean square deviation, Rku: kurtosis of the assessed profile, Rsk: skewness of the assessed profile and standard deviation are shown in Table 2.

### 2.4. Statistical Analysis

The software package IBM SPSS statistics 21.0 (Armonk, New York, NY, USA), was used to evaluate statistically significant differences between the groups, *p* < 0.05. Data, previously recorded in an Excel database, were submitted to one-way analysis of variance (ANOVA) followed by LSD post-hoc test.

## 3. Results

### 3.1. EDX Analysis

#### 3.1.1. Steel Drill

**Figure 3:**
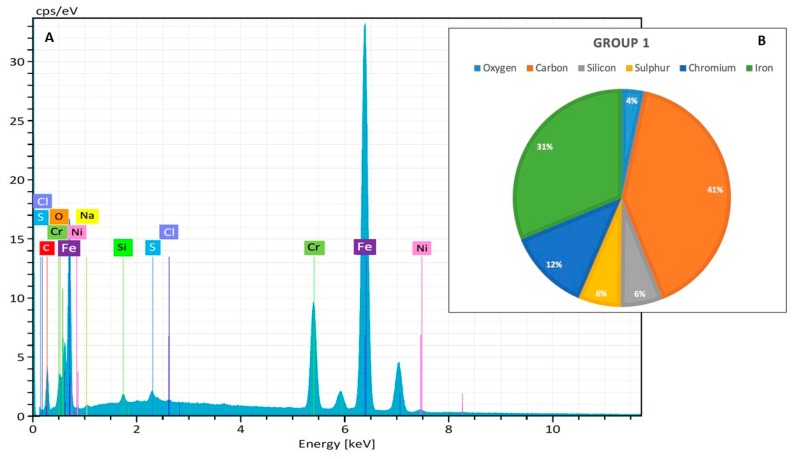
(**A**) The superficial composition of new zirconia drills, without any treatment (keV = kilo-electron-volt, accelerating voltage range used for EDX analysis; cps/eV: counts per second per electron-volt). (**B**) The atomic percentage (%), that represent the % concentration of each element on the same sample.

Group 1, the control, was characterized by more than 40% of the mass being carbon; the other elements particularly represented were chromium, iron, sulfur and silicon. Only a mild presence of oxygen was detected (4%) (Figure 3).

After the action of the decontaminants, the composition had changed:

Group 2 was composed of lower values of iron (12.00%) and chromium (5.30%) and an increased level of carbon (61.1%) and oxygen (10.19%) and similar percentages of sulfur (4.17%).

Group 3 was composed of an increased percentage of iron (38.53%), a mild increase of oxygen (8.05%) and sulfur (9.46%), similar levels of chromium (14.46%) and a lower percentage of carbon (26.19%).

Group 4 was composed of lower values of iron (10.22%) and chromium (5.16%), a similar percentage of sulfur (5.66%), an increased level of carbon (50.61%) and oxygen (20.71%).

In the test groups, there were great fluctuations in the levels of oxygen, carbon, chromium and iron. A contemporary increase of carbon and oxygen and the decrease of chromium and iron were detected in Groups 2 and 4. On the contrary, Group 3 was characterized by only a mild increase of oxygen, sulfur and chromium, an increase of iron and a decrease of carbon percentage.

#### 3.1.2. Zirconia Drill

**Figure 4:**
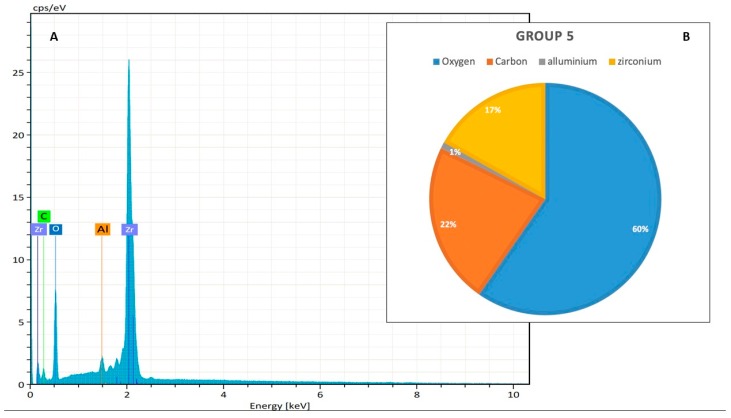
(**A**) The superficial composition of new SS drills without any treatment, by Energy-Dispersive X-ray Spectroscopy (EDX) analysis (keV = kilo-electron-volt, accelerating voltage range used for EDX analysis; cps/eV: counts per second per electron-volt). (**B**) The atomic percentage (%), that represent the % concentration of each element on the same sample.

Group 5, The EDX analysis showed that the new zirconia burs were composed mainly of oxygen (60%), zirconium (17%) and carbon (22%), (Figure 4).

Group 6, the effect of 50 cycles of decontamination with glutaraldehyde 2% were a mild decrease of zirconium and oxygen and a contemporary increase of carbon.

Group 7, a similar effect showed after decontamination with hydrogen peroxide 6%.

Group 8, the effect of 50 cycles of heat sterilization in autoclave was a mild decrease of zirconium and oxygen and a contemporary increase of carbon. The release of zirconium was about 4.00%.

### 3.2. Infrared Thermography

**Figure 5:**
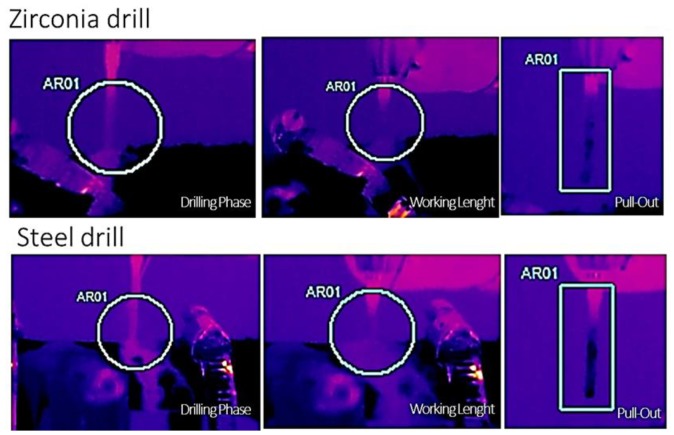
Real-time infrared thermography of the zirconia drill (Group 8) and stainless steel (Group 4). A detail of the temperature variations during the drilling phase, at working length and at drill pull-out.

Statistical analysis of infrared thermography of SS drills showed that Group 2—followed by Group 3—were characterized by a slight increase in temperature, during drilling, both in the apical portion rather than in the whole internal site, with significant differences in respect to the other groups analyzed in this study (*p* < 0.05) (Figure 5, Table 1). On the contrary, there were no significant differences among the different groups of zirconia drills concerning the temperature of the bone ribs, reached during drilling (Table 1).

### 3.3. Scanning Electron Microscopy (SEM)

**Figure 6:**
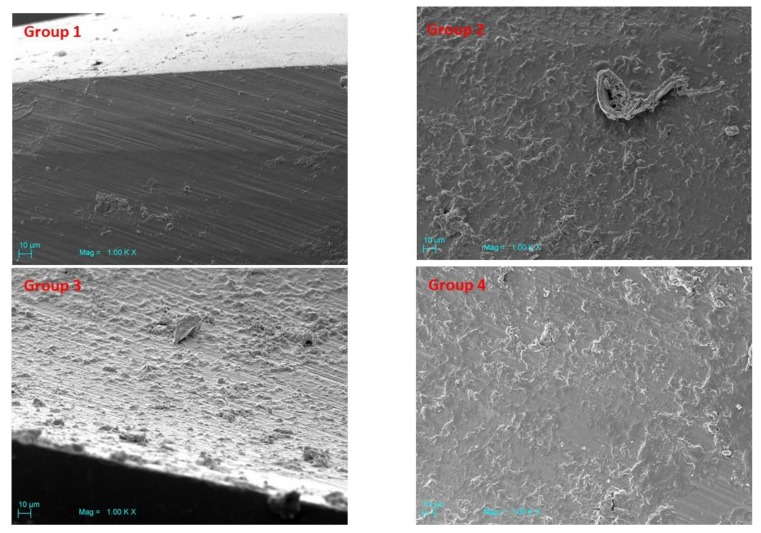
Scanning electron microscopy of Groups 2–4 showed the highest surface roughness vs Group 1. SS surfaces appeared covered with organic material.

**Figure 7:**
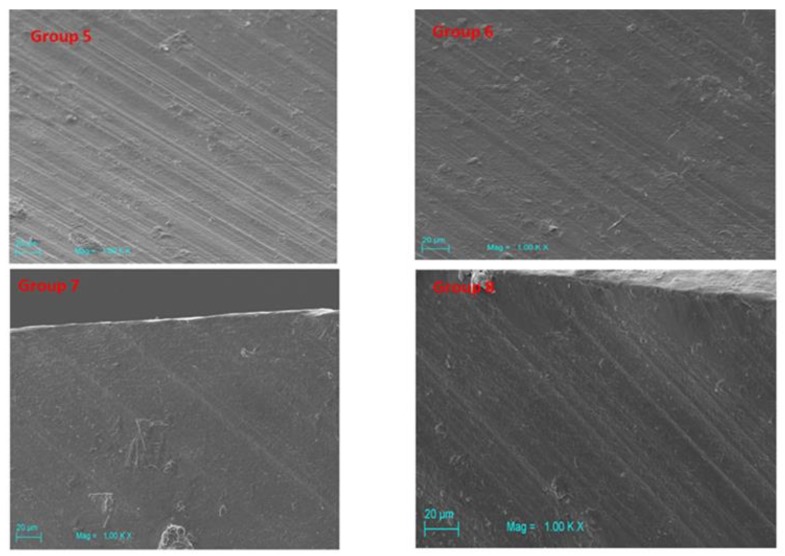
Zirconia drill Groups 5–8 showed more uniform regularities that were attributed mainly to the effect of fabrication.

**Figure 8:**
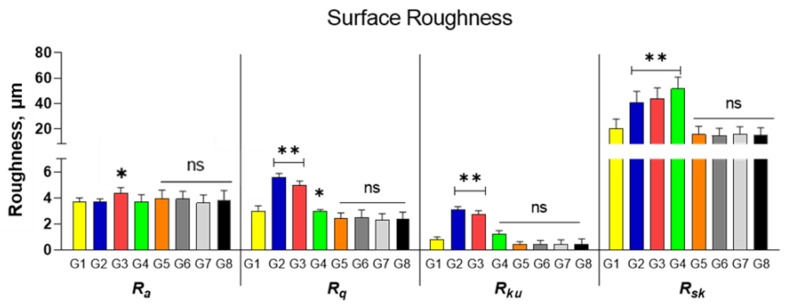
Surface roughness parameters (Ra, Rq, Rku and Rsk) of the study groups (mean, standard deviation). * *p* < 0.05, ** *p* < 0.01; n.s., Not significant.

Scanning electron microscopy (SEM) of Groups 2–4 showed a higher surface roughness vs Group 1. SS surfaces appeared covered with organic material (Figure 6). Zirconia drill Groups 5–8 showed more uniform regularities that were attributed mainly to the effect of fabrication (Figure 7). The 3D images of Groups 5–8 revealed the similarity of their surfaces, suggesting that liquid disinfection and heat sterilization processes have no influential effect on surface roughness. (Table 2, Figure 8) A little organic material was detected on the surface.

## 4. Discussion

Cross infections remain one of the major challenges in the dental profession, especially in oral surgery. Transmission of human immunodeficiency viruses, hepatitis B, hepatitis C and bovine spongiform encephalopathy are a major concern for dental staff and patients. These dangers can be eliminated by effective sterilization and disinfection techniques. In this research we used hydrogen peroxide and glutaraldehyde chemical disinfection for implant drills. It has been shown that repeated sterilization cycles cause undesirable changes in the physical properties of steel instruments, such as corrosion, temper shifts and loss of cutting sharpness [24].

Microscopical alterations of bur surfaces, due to galvanic electrochemical corrosion phenomena, are important issues, because of the risk of releasing metal particles into the tissues, during the implant bed preparation [25]. One of the major concerns of metal ions release is that they are of small size and for this reason they are ingested by macrophages, they can disseminate systemically and they could potentially cause cytotoxic, genotoxic and immunological effects, not only locally but also at distance from the implant site [25].

The presence of abundant irrigation is fundamental to control this problem [9]. Heat generation during the implant bed preparation can be influenced by drilling speed [26], sharpness of the cutting tool [27], drilling depth [28], drill geometry [28,29], use of internal or external irrigation [30], use of graduated versus one-step drilling [31], intermittent versus continuous drilling and drill material [32]. All these variables are controllable in clinical practice, while the pressure applied to the drill is not controllable. Also, the different densities of bone influence resistance to drilling with an increase of temperature [23,33]. However, the alteration of microscopical surfaces of the burs could affect their cutting ability, determining an increased heat generation during drilling. In-depth knowledge of superficial changes that characterize burs after a specific number of cycles of disinfection/sterilization, is fundamental to control these side effects that could cause early implant failure. Energy-dispersive X-ray spectroscopy (EDX) that uses X-rays emitted by the surface of a sample permitted us to analyze qualitatively and quantitatively the elemental composition of the burs after a variable number of cycles of disinfection. This technique was used to analyze the chemical distribution and bonding and to measure the elemental composition of the parts per thousand range, chemical state and empirical formula.

Group 2 was characterized by lower values of iron and chromium and increased levels of carbon and oxygen at EDX analysis; however, the infrared thermography recorded a higher temperature increment in respect to the controls of group1. Glutaraldehyde (1,5-pentanedial) has strong reducibility, relatively weak oxidation properties and exerts its antibacterial activity affecting the permeability of the cellular membrane. It is largely used because it is a non-oxidizing biocide [34,35]. However, the effect of a chemical sterilant depends on many factors, like the presence of organic and inorganic material, the pH, concentration, contact time and temperature.

Although there was a modest increment in oxygen and iron content in Group 3, the drills treated with hydrogen peroxide were characterized by a significant temperature increase during osteotomies. The corrosive effect of hydrogen peroxide on stainless steel (SS) has been largely demonstrated and the resultant extent of pitting, crevice or weld area corrosion are dependent on the different steel alloys and H_2_O_2_ solutions [36]. Indeed, considering the wide and varied alloy compositions present on the market, the SS resistance to corrosion is very variable and an index, the pitting resistance equivalent number (P.R.E.N.), is used to evaluate the corrosion resistance of SS [37]. The presence of Cr in the burs tested promoted the formation of Cr(OH)3/Cr_2_O_3_, a passive layer that is protective against localized corrosion promoted by hydrogen peroxide [38]. However, even with the presence of Cr, phenomena of localized corrosion is possible, due to the breakup of the layer of passivation [39]. The increment of iron percentage could increase the production of ROS that promotes osteoblastic apoptosis, a decreased differentiation and mineralization of osteogenic cells and affect the viability of bone marrow mesenchymal stem cells [40].

The EDX analysis showed that SS drills treated with 50 cycles of heat sterilization in autoclave were characterized by a higher increase of oxygen.

The limits regarding the accuracy of the infrared thermography technique are well-known by the authors, where the environmental measurements could be influenced by a random bias (ranged between 1–2%) determined by several aspects such as emissivity, reflected ambient temperature, transmittance, atmospheric temperature, camera response, large temperature gradients and calibrator accuracy [32,41,42].

In a laboratory-controlled environment—with stable conditions of temperature and humidity, in absence of large temperature gradients around the small areas of interest—these aspects were considered negligible for the present study purposes.

However, the infrared thermography found no significant differences in temperature increase between the study samples and controls. So, the thermal cycle, although increasing the oxygen content, did not affect the cutting ability of the drills. Steel drills are considered as suitable materials in dental implant bed preparation for oral rehabilitation; however, many biologic and physical events occur after osteotomy preparation [43]. One of the major reasons for these is the release of microparticles of steel, which threaten and induce reaction in bone or soft tissues. The results showed in the present study confirm those of a previous one [4]. However, this work adds further information regarding the effects of the disinfection agents on the drills. Indeed, we found a change of surface roughness on the SS drills and no effect on the zirconia drills.

Zirconia drills seem to not be particularly affected by the action of decontaminants; the levels of aluminum and zirconium remained constant: only a mild decrease of oxygen content (about 6.00%), with the contemporary increase of carbon (11.01%) was recorded. Indeed, tetragonal zirconia, stabilized with yttrium, is characterized not only by high biocompatibility by also by excellent mechanical properties, such as high strength, fracture resistance and wear behavior [44].

The results of the present study show that zirconia drills are more resistant to the corrosive action of disinfecting chemicals and the temperature produced during drilling did not change with the different treatments. The SEM and EDX analysis of drills subjected to different protocols permitted us to evaluate their impact on some characteristics of the drills, in particular, the drill point angle, drill material and wearing and drill geometry. Also, the use of infrared thermography permitted us to estimate the impact of these modifications on the temperature increase during implant site preparation [23].

It has been found that the surface roughness of steel drills is affected by the disinfection/sterilization protocols adopted. Another finding is the organic material detected on the SS surface. We do not know the effects that this residue has on bone healing or on the risk of cross-infection, however its role in cross-infection needs to be investigated.

## 5. Conclusions

Zirconia burs seem to be the most resistant against decontaminants; although Group 4 was characterized by a higher increase of superficial oxygen; it seems to be the safest treatment for SS burs concerning the increase of temperature during drilling. A statistically significant difference was observed for drilling time preparation and temperature between steel cylindrical drills and zirconia cylindrical drills (*p* < 0.01). The disinfection agents produce a slight impact on the temperature changes during bone preparation for implant placement, while heat sterilization processes have no effect on either type of drill used in the present study. The disinfection agents increase the roughness of steel drills, while there was no effect on the zirconia drills. Another discovery of this study is that zirconia is easier to clean which makes their use safer.

## Figures and Tables

**Table 1:** Summary of the cortical bone temperature after drilling site preparation (mean ± standard deviation) (* *p* < 0.05).

Bone Temperature (°C)				
**Stainless Steel Drill**	**Group 1**	**Group 2**	**Group 3**	**Group 4**
	38.92 ± 1.13	41.47 ± 1.06	41.51 ± 1.22	41.38 ± 1.15
**Zirconia Drill**	**Group 5**	**Group 6**	**Group 7**	**Group 8**
	**38.21 ± 1.01**	**38.9 ± 1.36**	**39.55 ± 1.79**	**39.43 ±0.98**
*p* value	*p* = 0.925	*p* = 0.041 (*)	*p* = 0.029 (*)	*p* = 0.037 (*)

**Table 2:** Summary of the surface roughness parameters of the study groups.

Surface Roughness _[μm]_	R_a_	R_q_	R_ku_	R_sk_
Group 1	3.671 ± 0.317	2.992 ± 0.391	0.832 ± 0.173	20.324 ± 7.413
Group 2	3.683 ± 0.236	5.592 ± 0.297	3.120 ± 0.214	40.683 ± 8.835
Group 3	4.341 ± 0.461	4.992 ± 0.313	2.720 ± 0.292	43.683 ± 8.591
Group 4	3.721 ± 0.532	3.001 ± 0.112	1.220 ± 0.261	51.683 ± 9.142
Group 5	3.945 ± 0.651	2.412 ± 0.437	0.4623 ± 0.169	15.648 ± 6.392
Group 6	3.912 ± 0.591	2.512 ± 0.554	0.4627 ± 0.271	14.623 ± 5.902
Group 7	3.622 ± 0.613	2.313 ± 0.471	0.4645 ± 0.308	15.661 ± 6.102
Group 8	3.845 ± 0.733	2.411 ± 0.494	0.4522 ± 0.405	15.111 ± 5.925

Ra: Arithmetical mean deviation; Rq: Root mean square deviation; Rku: Kurtosis of the assessed profile; Rsk: Skewness of the assessed profile.
